# A Compact and Efficient Boost Converter in a 28 nm CMOS with 90 mV Self-Startup and Maximum Output Voltage Tracking ZCS for Thermoelectric Energy Harvesting

**DOI:** 10.3390/s23136243

**Published:** 2023-07-07

**Authors:** Muhammad Ali, Seneke Chamith Chandrarathna, Seong-Yeon Moon, Mohammad Sami Jana, Arooba Shafique, Hamdi Qraiqea, Jong-Wook Lee

**Affiliations:** Information and Communication System-on-Chip (SoC) Research Center, School of Electronics and Information, Kyung Hee University, Yongin 17104, Republic of Korea

**Keywords:** thermoelectric generator, wearable electronics, boost converter, maximum power point tracking, zero current switching, self-startup

## Abstract

There are increasing demands for the Internet of Things (IoT), wearable electronics, and medical implants. Wearable devices provide various important daily applications by monitoring real-life human activities. They demand low-cost autonomous operation in a miniaturized form factor, which is challenging to realize using a rechargeable battery. One promising energy source is thermoelectric generators (TEGs), considered the only way to generate a small amount of electric power for the autonomous operation of wearable devices. In this work, we propose a compact and efficient converter system for energy harvesting from TEGs. The system consists of an 83.7% efficient boost converter and a 90 mV self-startup, sharing a single inductor. Innovated techniques are applied to adaptive maximum power point tracking (A-MPPT) and indirect zero current switching (I-ZCS) controllers for efficient operation. The startup circuit is realized using a gain-boosted tri-state buffer, which achieves 69.8% improved gain at the input *V*_IN_ = 200 mV compared to the conventional approach. To extract the maximum power, we use an A-MPPT controller based on a simple capacitive divider, achieving 95.2% tracking efficiency. To address the challenge of realizing accurate voltage or current sensors, we propose an I-ZCS controller based on a new concept of maximum output voltage tracking (MOVT). The integrated circuit (IC) is fabricated using a 28 nm CMOS in a compact chip area of 0.03 mm^2^. The compact size, which has not been obtained with previous designs, is suitable for wearable device applications. Measured results show successful startup operation at an ultralow input, *V*_IN_ = 90 mV. A peak conversion efficiency of 85.9% is achieved for the output of 1.07 mW.

## 1. Introduction

Recent interest in renewable energy sources is significantly increasing with rising environmental pollution and energy costs. Extensive research has been conducted to explore new methods to conserve power and harvest clean energy. Because of the increasing number of low-power devices such as the Internet of Things (IoT), wearable electronics, and medical implants, energy harvesting from renewable sources is an urgent research topic [1]. Among them, wearable devices provide important daily applications by monitoring various human activities. Diverse examples of wearable devices have been proposed, which include electronic skin, wristbands, watches, smart clothing, virtual reality (VR) headsets, and artificial intelligence (AI) hearing aids [2]. These devices demand low-cost autonomous operation with miniaturized form factors; the demands are challenging to satisfy using a rechargeable batteries. One promising energy source for wearable electronics is thermoelectric generators (TEGs), which convert an omnipresent temperature gradient into electrical energy via the Seebeck effect [3,4]. It is considered that TEGs are the only way to generate a small amount of electric power for the autonomous operation of wearable devices. Because energy conversion can be performed using a solid-state material without moving elements, TEGs are suitable for long-term operation. Under a relatively small temperature gradient, however, TEG generates a low source voltage (*V*_S_), which cannot be used to supply the devices directly. For example, commercially available TEGs generate *V*_S_ between 10 mV and 30 mV for a 1 °C temperature difference [5]. Therefore, a DC-DC converter is required to boost the input to a suitable output. 

Various techniques have been explored to realize efficient DC-DC converters using maximum power point tracking (MPPT) methods [6,7,8,9]. The authors of [6] configure the switching frequency based on the internal resistor (*R*_S_) of the TEG and the inductor value. However, the frequency of the on-chip clock generator can fluctuate due to process, voltage, and temperature (PVT) variations. This necessitates an adaptive MPPT controller based on a voltage-controlled oscillator (VCO) to tune the switching frequency [7]. The work conducted in [8] utilizes a hill-climbing MPPT controller that senses the output current of the converter, which charges algorithm capacitors to track *V*_S_ for handling the input range from 5 μW to 10 mW. The work conducted in [9] uses transformer-based methods for a dual-stage boost converter to increase conversion efficiency to 81.5%.

The switched capacitor (SC)-based approach offers the advantage of on-chip integration and low-voltage operation [10,11,12]. The gate-boosted charge pump operates with an input voltage of 100 mV [10]. Another work [11] utilized a voltage doubler as a negative charge pump to reduce the on-resistance of the load-side switch. Additionally, a fully integrated SC converter used a 220 mV input to generate a 1.9 V output [12]. However, previous works have the drawback of large system sizes; for example, the algorithm capacitor in [8] requires a 7.6 mm^2^ silicon area. Other works have bulky implementations, such as a transformer of 1.8 mm^2^ size [9], a sizeable on-chip capacitor of 2.31 mm^2^ [10], and three inductors [13]. The SC-based converter shows relatively low efficiencies, for example, 33% [10], 52% [11], and 37.4% [12].

Because *V*_S_ can be smaller than the threshold voltage (*V*_TH_) of transistors, a self-startup method is required for the initial step-up of the supply voltage. The startup temporarily provides power for the main converter until it can be self-sustained. Various methods have been investigated. These methods include using a mechanical switch to initiate the converter [6], utilizing an off-chip transformer [9], using on-chip inductors [10], and pre-charging the output capacitor using an external battery [14]. However, these techniques often demand bulky implementations, which can be impractical for low-cost sensor nodes and IoT applications. The works [15,16] use the switched branches for inductor sharing between the startup and main converter; however, the switching increases the system complexity. 

The discontinuous conduction mode (DCM), commonly used for low-power DC-DC converters, should suppress the reverse current through the inductor. The high-side switch is opened when the inductor current *I*_IND_ is close to zero for zero current switching (ZCS). One method to implement ZCS is using a diode [17]; however, this approach is unsuitable for low-voltage design due to diode voltage drop. For efficiency reasons, a transistor switch is typically used with a ZCS controller. A previous work on a ZCS controller used a comparator to detect the voltage [18]. Another approach is to use digital circuits to detect current zero crossing indirectly using the inductor voltage [19]. The digital quantization of inductor voltage needs to select fine pulse width for the ZCS operation. However, the linear scaling used to modulate the pulse width becomes inefficient for low *V*_IN_. To handle the issue, a fine delay stage is used [20], which sets the pulse width suitable to open the high-side switch; however, the ZCS controller uses an external supply to realize fine delay. In addition, appropriate measurement delay is not addressed [21].

To overcome the issues mentioned above, we propose a compact and efficient DC-DC converter system realized in the 28 nm CMOS technology. The integrated system consists of an 85.9% efficient boost converter and a 90 mV self-startup, sharing a single inductor. Innovated techniques are applied to adaptive MPPT (A-MPPT) and indirect ZCS (I-ZCS) controllers for efficient operation. The startup circuit is realized using a ring-oscillator-based charge pump, which enhances gain using a tri-state buffer for ultralow voltage operation. The tri-state buffer achieves a 69.8% gain improvement over a single buffer at *V*_IN_ = 200 mV. To extract the maximum power, we use an A-MPPT controller based on a simple capacitive divider for fractional open circuit voltage (FOCV) operation. To address the challenges of realizing accurate voltage or current sensors, we propose an I-ZCS controller based on maximum output voltage tracking (MOVT). The fabricated integrated circuit (IC) is realized in a compact chip area of 0.03 mm^2^. The compact size, which has not been obtained with previous designs, is suitable for wearable device applications. The measured results show a peak tracking efficiency of 95.2% and a conversion efficiency of 85.9%. The end-to-end efficiency of 83.7% is achieved using *R*_S_ = 8 Ω and *V*_S_ = 280 mV. 

This paper is organized as follows. Section 2 describes the system design. Section 3 presents the circuit implementation. Section 4 presents the measured results, and Section 5 draws the conclusion.

## 2. System Design

### 2.1. Proposed System

**Figure 1:**
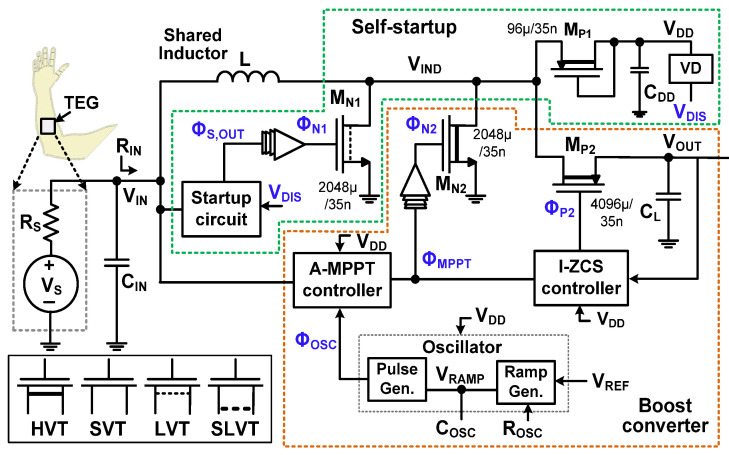
Block diagram of the proposed system.

Figure 1 shows the block diagram of the proposed system. It mainly consists of a boost converter and a self-startup circuit. The boost converter includes an A-MPPT controller, an I-ZCS controller, a voltage detector (VD), an oscillator, and four power switches (M_N1_, M_N2_, M_P1_, and M_P2_). M_N1_, used for self-startup, is a low threshold voltage (LVT) transistor. High threshold voltage (HVT) transistors are used for the switches of the boost converter (M_N2_, M_P1_, and M_P2_), which reduces the leakage current compared to the standard threshold voltage (SVT) transistor. Super-low threshold voltage (SLVT) transistors are used for the startup circuit to reduce the minimum input *V*_IN_ to the system. The oscillator consists of a ramp generator, pulse generator, biasing resistor *R*_OSC_, and capacitor *C*_OSC_. The ramp voltage *V*_RAMP_ is compared with a reference *V*_REF_ to generate the clock signal Φ_OSC_ for the A-MPPT controller. The TEG is modeled using *V*_S_ and *R*_S_. An input capacitor *C*_IN_ is used to buffer the converter’s input *V*_IN_. A single inductor *L* = 100 μH is shared between the startup and boost converter without extra switches. 

**Figure 2:**
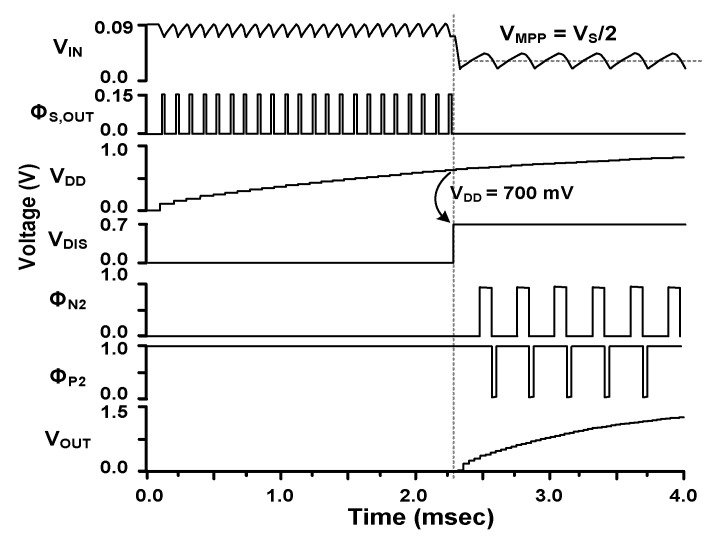
Waveforms of the converter operation.

Figure 2 shows the waveform of the converter. When *V*_IN_ is supplied to the startup circuit, it generates an output pulse Φ_S,OUT_ (=Φ_N1_) for M_N1_. During the on-time of M_N1_, *L* is charged when *V*_IN_ is as low as 90 mV. During the off-time of M_N1_, stored energy in *L* keeps charging the storage capacitor *C*_DD_ through M_P1_. During the startup, the supply voltage *V*_DD_ gradually increases to 700 mV. The VD monitors *V*_DD_. When *V*_DD_ reaches 700 mV, it generates the disable signal *V*_DIS_, which stops the oscillation of the startup circuit and enables the boost converter. The boost converter raises the tens of mV generated by the TEG to the output *V*_OUT_ level required to power IoT devices. To extract the maximum power from the TEG, the A-MPPT controller continuously adjusts the on-time of the pulse Φ_N2_ for M_N2_. Then, the input impedance *R*_IN_ is adjusted to match *R*_S_, and the maximum power point (MPP) voltage *V*_MPP_ tracks half of V_S_. The I-ZCS controller provides the pulse Φ_P2_ to control the on-time of M_P2_ when *L* discharges. The M_P2_ is turned off at a suitable time to prevent the current backflow from *V*_OUT_.

### 2.2. Self-Startup Circuit

Ring oscillators are widely used to generate the clock for the startup circuit; however, the operation is limited mainly by two reasons. Firstly, when V_DD_ reduces, it is difficult to sustain the oscillation with the decreased gain of the inverter. Secondly, the output swing needed to drive the subsequent stage is reduced. These limitations can significantly impede the low-voltage startup [22]. 

**Figure 3:**
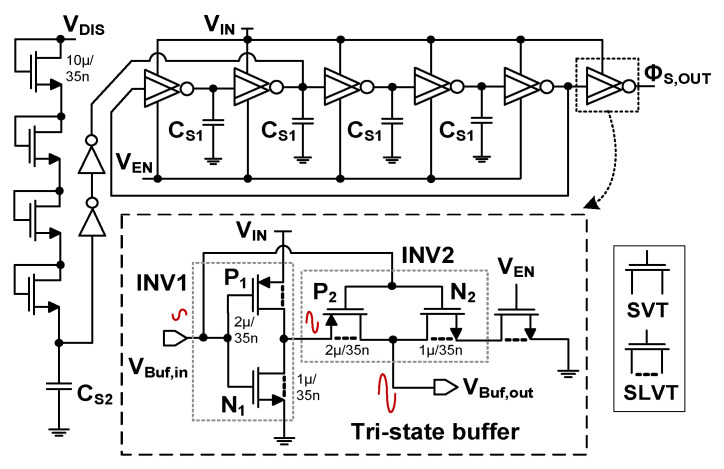
Schematic of the proposed self-startup circuit using the gain-boosted tri-state buffer. *C*_S1_ *=* 100 fF; *C*_S2_ = 1.5 pF.

Figure 3 shows the proposed self-startup circuit using a gain-boosted tri-state buffer. It consists of six stages, in which the output of the fifth stage is feedback to the first stage. The inset shows the schematic of the unit stage. It comprises two inverters, INV1 (transistors N_1_ and P_1_) and INV2 (transistors N_2_ and P_2_). They are realized using SLVT transistors to reduce the startup voltage further. The INV2 works as a tri-state buffer, which provides additional gain to reduce the input for the startup. *V*_EN_ is enabled by the input, and the startup circuit generates Φ_S,OUT_. When disabled, it keeps a high impedance state. The voltage gain *A*_V1_ of a single inverter can be expressed [22] as

(1) $$A_{V1}≅\frac{g_{m,\mathrm{eff}1}}{g_{\mathrm{ds},N1}+g_{\mathrm{ds},P1}}=\frac{g_{m,N1\;}+g_{m,P1\;}}{g_{\mathrm{ds},N1}+g_{\mathrm{ds},P1}}$$

where the effective transconductance *g*_m,eff1_ is the transconductance sum of N_1_ and P_1_. The *g*_ds,N1_ and *g*_ds,P1_ are the output conductance of N_1_ and P_1_, respectively. In the proposed gain-boosted buffer, the input *V*_Buf,in_ is fed to the gate of the two inverters. The buffer output *V*_Buf,out_ is taken from the output of INV2. The output of INV1 is connected to the source of the P_2_, which is amplified by the gain (*A*_V1_*V*_IN_) of INV1. Then, it is increased to (1 + *A*_V1_)*V*_IN_. Because *A*_V1_ > 1, *V*_Buf,in_ is amplified by the effective transconductance *g*_m,eff2_ of the tri-state buffer, which can be written as

(2) $$g_{m,\mathrm{eff}2}≅(1+A_{V1})g_{m,P2}+g_{m,N2}$$

where *g*_m,P2,_ and *g*_m,N2_ are the transconductance of P_2_ and N_2_, respectively. Using a similar method to that used to derive Equation (1), we obtain the gain *A*_V2_ of the gain-boosted buffer as

(3) $$A_{V2}≅\frac{g_{m,\mathrm{eff}2\;}}{g_{\mathrm{ds},P2}+g_{\mathrm{ds},N2}}=\frac{(1+A_{V1})g_{m,P2\;}+g_{m,N2\;}}{g_{\mathrm{ds},P2}+g_{\mathrm{ds},N2}}$$

where *g*_ds,N2,_ and *g*_ds,P2_ are the output conductance of N_2_ and P_2_, respectively. The result shows an enhanced gain of the tri-state buffer. 

**Figure 4:**
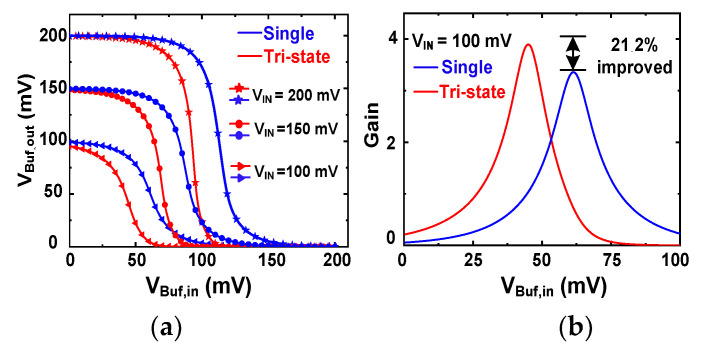

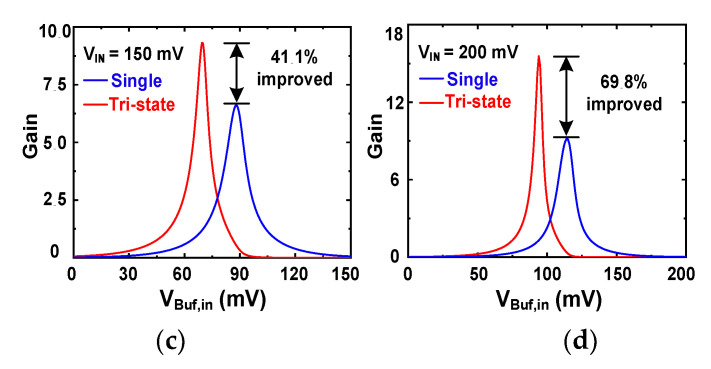
(**a**) Transfer characteristics of the single inverter and the gain-boosted tri-state buffer. Gain comparison at three input values of (**b**) *V*_IN_ = 100 mV, (**c**) *V*_IN_ = 150 mV, and (**d**) *V*_IN_ = 200 mV.

Figure 4a shows the transfer characteristics of the single- and tri-state buffers for three *V*_IN_. The tri-state buffer shows steeper slopes, indicating increased gain. The gain is calculated by taking the derivative of *V*_Buf,out_. Figure 4b shows the gain comparison using *V*_IN_ = 100 mV. The result indicates that the tri-state buffer achieves a 21.2% gain improvement. Figure 4c,d show the gain comparison using *V*_IN_ = 150 mV and *V*_IN_ = 200 mV, respectively. The results show that the tri-state buffer increases the gain by 41.1% and 69.8%, making it an attractive approach for low-voltage startups.

### 2.3. Loss Analysis for Startup

**Figure 5:**
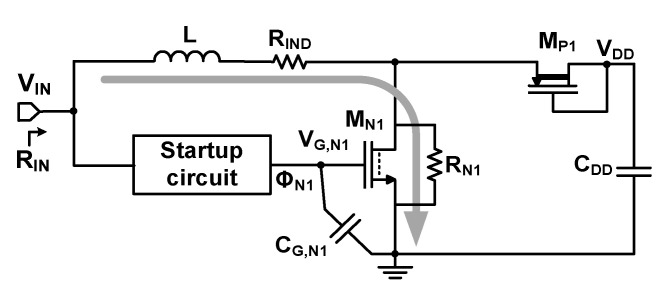
Equivalent circuit of the startup circuit for loss analysis.

Figure 5 shows the equivalent circuit of the startup for loss analysis. The resistive power loss *P*_R,startup_ of the startup circuit can be expressed as

(4) $$P_{R,\mathrm{startup}}≅\frac{R_{0}V_{\mathrm{IN}}^{2}}{{\left(R_{\mathrm{IN}}+R_{0}\right)}^{2}}=\frac{(R_{\mathrm{IND}}+R_{N1})V_{\mathrm{IN}}^{2}}{{\left(R_{\mathrm{IN}}+R_{\mathrm{IND}}+R_{N1}\right)}^{2}}$$

where *R*_0_ consists of the inductor parasitic resistance *R*_IND_ (= 120 mΩ) and the resistance *R*_N1_ of M_N1_. The switching loss *P*_SW,startup_ can be expressed as

(5) $$P_{\mathrm{SW},\mathrm{startup}}≅\frac{1}{2}C_{G,N1}V_{G,N1}^{2}f_{\mathrm{St}}$$

where *f*_St_ is the switching frequency of the startup circuit. *V*_G,N1_ and *C*_G,N1_ are the gate voltage and capacitance of M_N1_, respectively. The power consumption *P*_Q,startup_ of the startup circuit is 60 nW using *V*_S_ = 150 mV and *R*_S_ = 18 Ω. The total loss *P*_T,startup_ is the sum of *P*_R,startup_, *P*_SW,startup_, and *P*_Q,startup_. Using the input power *P*_IN_, we obtain the efficiency *η*_startup_ of the startup circuit as

(6) $$\eta_{\mathrm{startup}}=\frac{P_{\mathrm{IN}}-P_{T,\mathrm{startup}}}{P_{\mathrm{IN}}}$$

**Figure 6:**
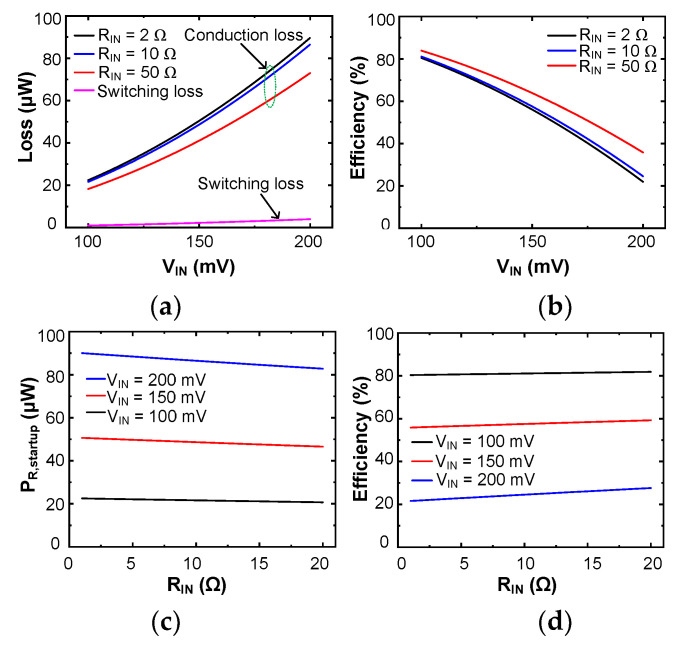
Calculated loss and efficiency of the startup circuit: (**a**) power loss, (**b**) efficiency as a function of *V*_IN_ for different *R*_IN_, (**c**) conduction loss, and (**d**) efficiency as a function of *R*_IN_ for different *V*_IN_.

Figure 6a shows the calculated loss of the startup circuit as a function of *V*_IN_. *P*_R,startup_ increases with *V*_IN_, and *P*_SW,startup_ is negligible compared to *P*_R,startup_ with a relatively low *f*_St_ ≈ 40 kHz. Figure 6b shows that *η*_startup_ decreases when *V*_IN_ increases for a given *R*_IN_. Figure 6c shows that *P*_R,startup_ decreases slightly with *R*_IN_, which is mainly determined by *V*_IN_. Figure 6d shows that *η*_startup_ increases with *R*_IN_ while decreasing with *V*_IN_, which is attributed to increased *P*_R,startup_. In summary, increasing *V*_IN_ leads to elevated *P*_R,startup_ and reduced *η*_startup_. Increasing *R*_IN_ results in reduced *P*_R,startup_ and improved *η*_startup_.

### 2.4. Loss Analysis for Boost Converter

**Figure 7:**
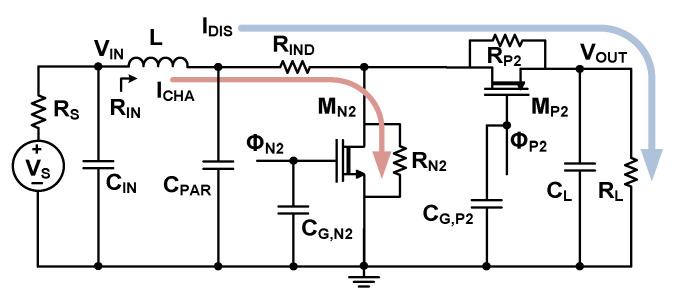
Equivalent circuit of the boost converter for loss analysis. *C*_IN_ = 20 nF, *R*_IND_ = 0.12 Ω, *C*_L_ = 0.5 μF, and *R*_L_ = 500 kΩ.

Figure 7 shows the equivalent circuit of the converter for loss analysis. The resistive power loss *P*_R,boost_ of the boost converter can be written as

(7) $$P_{R,\mathrm{boost}}={\left(\bar{I_{\mathrm{CHA}}}\right)}^{2}(R_{\mathrm{IND}}+R_{N2})+{\left(\bar{I_{\mathrm{DIS}}}\right)}^{2}(R_{\mathrm{IND}}+R_{P2})$$

where $\bar{I_{CHA}}$ is the average inductor charging current when M_N2_ is on, and $\bar{I_{DIS}}$ is the average discharging current when M_P2_ is on (M_N2_ is off). *R*_N2_ and *R*_P2_ are the on-resistances of M_N2_ and M_P2_, respectively. In the typical boost converter, we can neglect the loss of M_P2_ with the condition of ($\bar{I_{CHA}}$ ≫ $\bar{I_{DIS}}$). The switching loss *P*_SW,boost_ can be expressed as

(8) $$P_{\mathrm{SW},\mathrm{boost}}=\frac{1}{2}\left[(C_{G,N2}+C_{\mathrm{PAR}})V_{\mathrm{DD}}^{2}+C_{G,P2}{\left(V_{\mathrm{OUT}}-V_{\mathrm{TH},P2}\right)}^{2}\right]f_{S}≅\frac{1}{2}\left(C_{G,N2}+C_{G,P2}+C_{\mathrm{PAR}}\right)V_{\mathrm{DD}}^{2}f_{S}$$

where *f*_S_ is the switching frequency, and we use the approximated relationship of *V*_DD_ ≈ (*V*_OUT_ – *V*_TH,P2_). Here, *V*_TH,P2_ = 0.52 V is the threshold voltage of M_P2_. *C*_G,N2_ and *C*_G,P2_ are the gate capacitance of M_N2_ and M_P2_, respectively. *C*_PAR_ = 21 pF is the parasitic capacitance. We obtain *P*_SW,boost_ = 200 nW using *f*_S_ = 8 kHz, and *V*_DD_ = 0.9 V. The quiescent power *P*_Q,boost_ of the boost converter is 420 nW. The total power loss *P*_T,boost_ of the boost converter is the sum of *P*_R,boost_, *P*_SW,boost_, and *P*_Q,boost_. The synchronization and leakage losses are neglected to simplify the analysis. Then, we obtain the end-to-end efficiency *η*_EE_ of the boost converter as

(9) $$\eta_{\mathrm{EE}}=\frac{P_{\mathrm{OUT}}}{P_{\mathrm{Max}}}=\frac{P_{\mathrm{Max}}-P_{T,\mathrm{boost}}}{P_{\mathrm{Max}}}$$

where *P*_Max_ = (*V*_S_)^2^/(4*R*_S_) is the maximum available power from the source, and *P*_OUT_ = (*V*_OUT_)^2^/*R*_L_ is the output power with the load resistance *R*_L_. 

**Figure 8:**
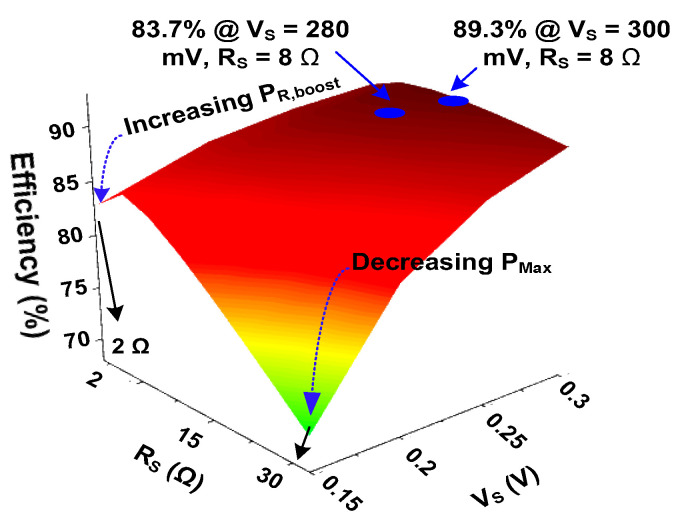
Calculated efficiency of the converter as a function of *V*_S_ and *R*_S_.

Figure 8 shows the calculated *η*_EE_ as a function of *R*_S_ for different *V*_S_ using the switch parameters shown in Table 1. When *R*_S_ is reduced, increased $\bar{I_{CHA}}$ and *P*_R,boost_ reduces *η*_EE_. The *η*_EE_ improves with increasing *R*_S_ for a given V_S_. For *R*_S_ = 8 Ω and *V*_S_ = 300 mV, *η*_EE_ increases to 89.3%. Beyond the maximum point, increasing *R*_S_ reduces *η*_EE_ by the decreased *P*_Max_.

## 3. Implementation

### 3.1. Adaptive MPPT Controller

**Figure 9:**
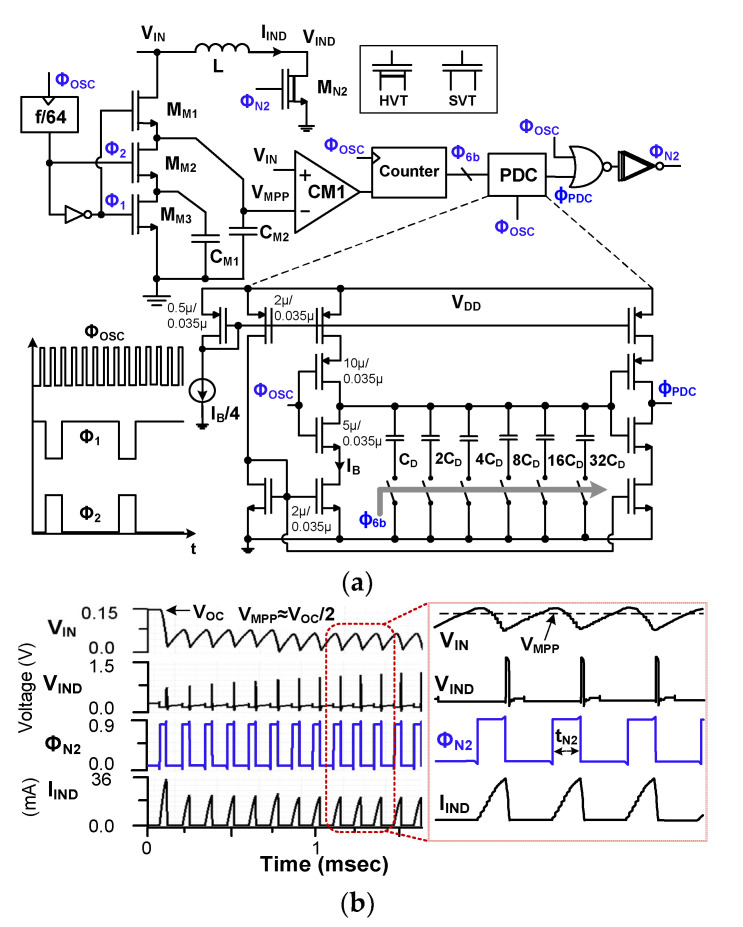
(**a**) Schematic of the A-MPPT controller; (**b**) simulated waveforms. *C*_D_ = 80 fF; C_M1_ = C_M2_ = 1 nF.

Figure 9a shows the schematic of the A-MPPT controller. It consists of a capacitor-based FOCV sampler, a comparator CM1, a counter, a programmable delay controller (PDC), and logic gates. In the FOCV technique, *V*_MPP_ ≈ (*α*_MPP_*V*_OC_) is obtained using the sampled open-circuit voltage *V*_OC_ (≈*V*_S_) and a source-dependent coefficient *α*_MPP_, where *α*_MPP_ = 0.5 is used for the TEG. The capacitive divider (*C*_M1_ and *C*_M2_) generates *V*_MPP_. When Φ_1_ is high (Φ_2_ is low), switch M_M1_ is turned on, and *V*_OC_ is stored in *C*_M2_. When Φ_2_ is high (Φ_1_ is low), switch M_M2_ is turned on. The stored charge in C_M2_ is shared with C_M1_. Then, the voltage *V*_MPP_ across *C*_M2_ becomes half of *V*_OC_. CM1 compares *V*_IN_ against *V*_MPP_, which decides the direction of the up/down counter. The counter output sets the delay of the PDC, and the on-time *t*_N2_ of Φ_N2_ is adjusted by the capacitor array in the PDC.

The *R*_IN_ of the DCM operation can be expressed [9] as

(10) $$R_{\mathrm{IN}}=\frac{2L}{t_{N2}^{2}f_{S}}$$

where we vary *t*_N2_ to adjust *R*_IN_ while keeping *f*_S_ constant. To facilitate the MPPT operation, the oscillator frequency *f*_OSC_, which is higher than *f*_S_, is used to update *t*_N2_. This method is different from a previous approach [23], which adjusts *t*_N2_ after evaluating *V*_MPP_ rather than choosing between different *V*_MPP_ values. Figure 9b shows the simulated waveforms of the MPPT operation. In the beginning, *V*_IN_ increases to *V*_OC_ by the sampling operation. Using the output of CM1, the A-MPPT controller adjusts *t*_N2_ to vary *R*_IN_ for tracking the *V*_IN_ changes occurring between two consecutive *V*_MPP_ values. The A-MPPT controller consumes 430 nW at *V*_DD_ = 900 mV. 

**Figure 10:**
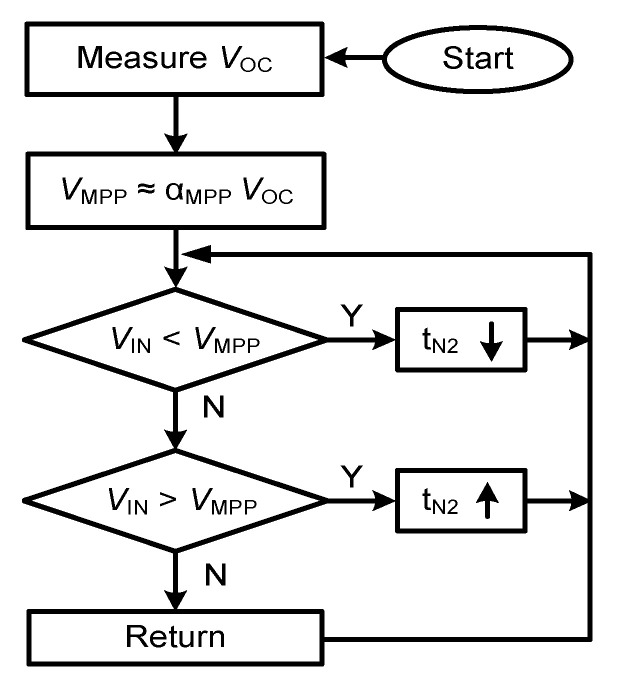
Flowchart of the MPPT operation.

**Figure 11:**
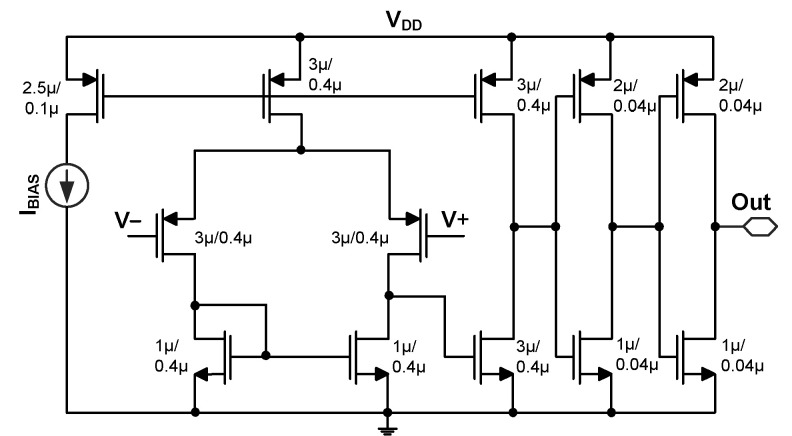
Schematic of the comparator.

Figure 10 shows the flowchart further explaining the MPPT operation. The operation is based on the approximately linear relationship between *V*_OC_ and *V*_MPP_. By leveraging this relationship, *t*_N2_ is adjusted to control *R*_IN_ and *V*_IN_. In the case of (*V*_IN_ < *V*_MPP_), *t*_N2_ is decreased to increase *R*_IN_ and *V*_IN_. Conversely, *t*_N2_ increases to reduce *R*_IN_. The proposed approach is simple since it requires measurements of only *V*_OC_, even during temperature variations. Figure 11 shows the schematic of CM1. It is based on a PMOS differential input pair and is biased, using *I*_BIAS_ = 5 nA. The power consumption of CM1 is 40 nW at *V*_DD_ = 0.9 V.

### 3.2. Indirect ZCS Controller

**Figure 12:**
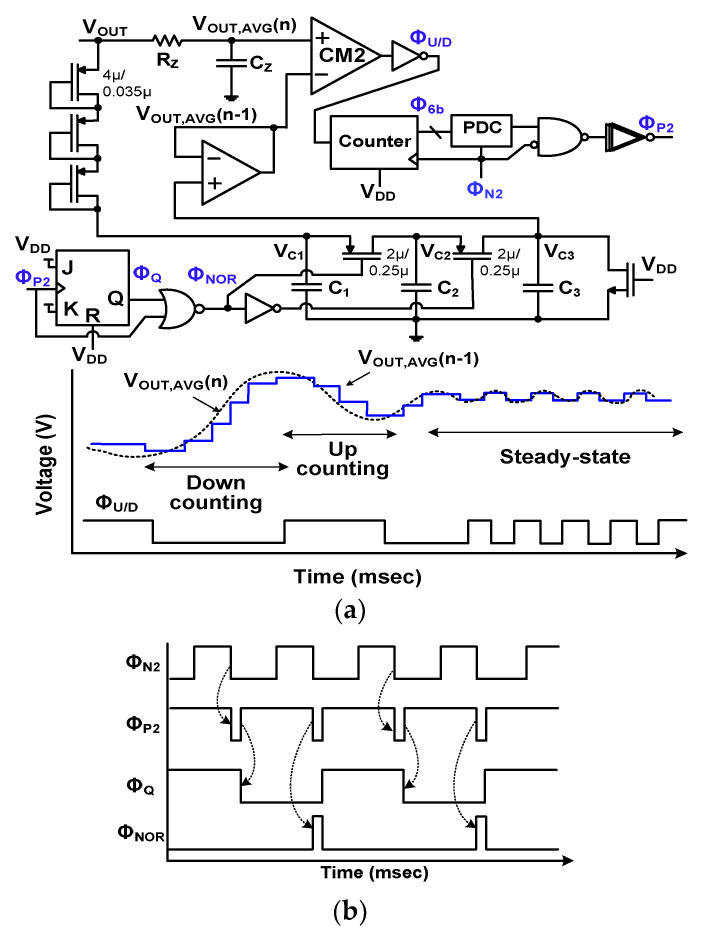
(**a**) Schematic of the I-ZCS controller; (**b**) logic operation. *C*_Z_ = 5 fF; *R*_Z_ = 36 kΩ; *C*_1_ = *C*_2_ = *C*_3_ **=** 16 fF.

Figure 12a shows the schematic of the I-ZCS controller. It comprises a comparator CM2, a counter, a PDC, sampling capacitors (*C*_1,2,3_), and logic gates. The CM2 compares the current average output *V*_OUT,AVG_(*n*) against the previous output *V*_OUT,AVG_(*n*−1), which is obtained via the low pass filter (*R*_Z_, *C*_Z_). The CM2 outputs the up/down signal Φ_U/D_ for the counter, followed by the PDC that generates the adjustable delay Φ_P2_ for M_P2_. Using the 6-b output Φ_6b_, PDC either increases or decreases the on-time *t*_P2_ of Φ_P2_. The PDC used for the I-ZCS controller has a similar structure to the one used in the A-MPPT controller. The Φ_N2_ is input to the PDC to set the starting reference for Φ_P2_ since the on-time of Φ_N2_ is used for inductor charging, while the on-time of Φ_P2_ is used for inductor discharging. At the end of discharging, the ZCS operation is performed. 

Using the waveform of *I*_IND_, we can derive the expression for the change Δ*t*_P_ in terms of output change Δ*V*_OUT_ = [*V*_OUT,AVG_(*n*) − *V*_OUT,AVG_(*n*−1)] as

(11) $$\left|\Delta t_{P2}\right|≅\left(\frac{L}{t_{N2}R_{\mathrm{OUT}}f_{S}}\right)\frac{\left|\Delta V_{\mathrm{OUT}}\right|}{V_{\mathrm{OUT}}}$$

where Δ*t*_P2_ is the difference between the current *t*_P2_(*n*) and the previous *t*_P2_(*n* − 1). The derivation of the above relation is shown in Appendix A. The nomenclature used for the acronyms and symbols of this paper are listed in Appendix B. The working principle of the I-ZCS controller is based on MOVT. *V*_OUT_ is maximized when optimal *t*_P2_ is set for ZCS. In either case, when *I*_IND_ falls to zero before or after M_P2_ is switched off, the non-optimal ZCS operation reduces *V*_OUT_; when there is perfect inductor zero crossing, *V*_OUT_ is maximized. One advantage of an I-ZCS operation is that no *I*_IND_ sensor is needed. The *t*_N2_ of Φ_N2_ is determined by the A-MPPT controller, depending on the source impedance *R*_S_. The *t*_N2_ does not have a direct effect on the I-ZCS controller since it is independently determined before the ZCS operation.

Waveforms for explaining I-ZCS operation are also shown. When *V*_OUT,AVG_(*n*) is greater than *V*_OUT,AVG_(*n*−1), the positive difference (Δ*V*_OUT_ > 0) performs down-counting with low Φ_U/D_. The PDC reduces the number of active delay capacitance, decreasing the pulse width of *t*_P2_. Conversely, the opposite condition (Δ*V*_OUT_ < 0) performs up-counting to increase *t*_P2_. Then, Φ_U/D_ switches between high and low until it reaches the steady state, when *V*_OUT_ is maximized by optimal ZCS operation. One drawback of this approach is that *V*_OUT_ is not fully regulated, which can be handled using additional regulators commonly used for various power supplies.

**Figure 13:**
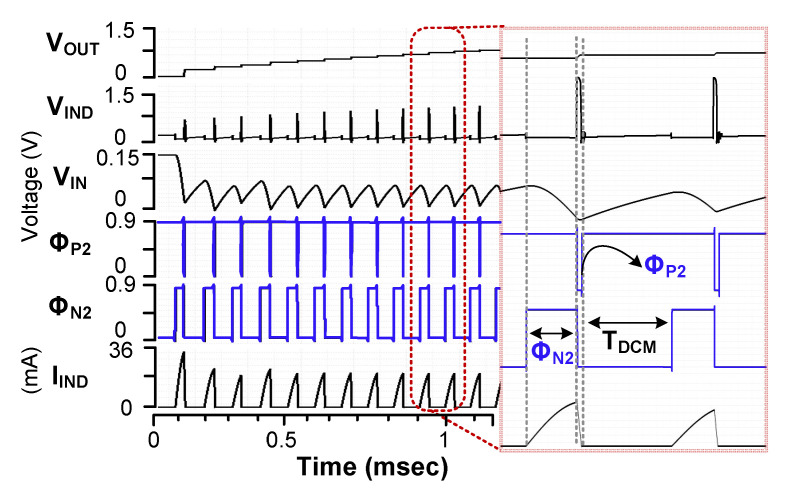
Simulated waveforms of the I-ZCS controller.

Figure 12b shows the logic operation to generate *V*_OUT,AVG_(*n* − 1). After Φ_N2_ is turned off, Φ_P2_ turns on M_P2_. The output Φ_Q_ is kept high until Φ_P2_ becomes high. With a high transition of Φ_P2_, the output Φ_Q_ of the J/K flip-flop becomes low. When Φ_P2_ becomes low, Φ_NOR_ becomes high, which allows *C*_2_ to be charged from *C*_1_. When Φ_NOR_ changes to low due to the rising Φ_P2_, the voltage *V*_C2_ across *C*_2_ is transferred to *C*_3_, generating *V*_OUT,AVG_(*n* − 1). Figure 13 shows the simulated results of the I-ZCS controller. During the on-time of Φ_N2_, *I*_IND_ increases. When Φ_N2_ turns off and Φ_P2_ turns on, *I*_IND_ decreases. The result shows that Φ_P2_ accurately switches off when the zero crossing of *I*_IND_ occurs. When *I*_IND_ reaches zero, M_P2_ turns off, initiating the DCM period indicated by *T*_DCM_. The result shows the successful operation of the proposed I-ZCS controller. 

## 4. Measured Results

**Figure 14:**
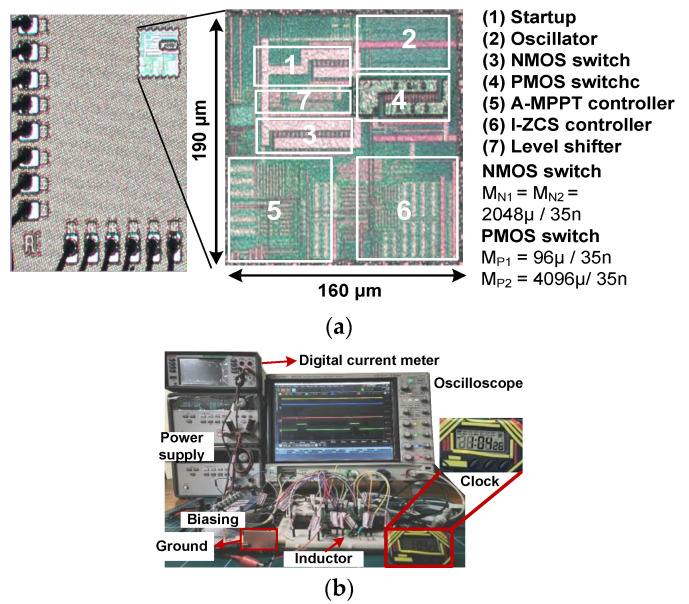
(**a**) Micrograph of the fabricated converter IC; (**b**) experimental setup.

Figure 14a shows the micrograph of the converter IC fabricated using a 28 nm CMOS process in an area of 0.03 mm^2^. The IC is mounted on a test board using the chip-on-board (COB) technique. Figure 14b shows the experimental setup for the converter characterization. A digital meter (DMM6500) is used for measuring the current. The DC power supply emulates the TEG source from 0.1 V to 0.4 V. The commercial TEG source is also used for testing [24].

**Figure 15:**
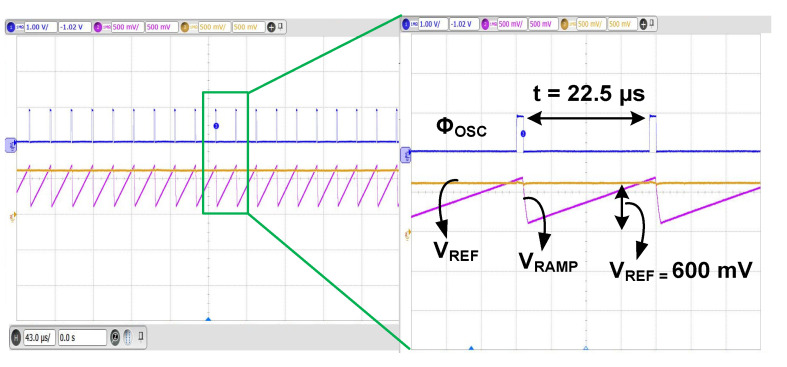
Measured waveforms of the oscillator. *C*_OSC_ = 22 pF. *R*_OSC_ = 100 kΩ is used to set the bias current of the oscillator.

**Figure 16:**
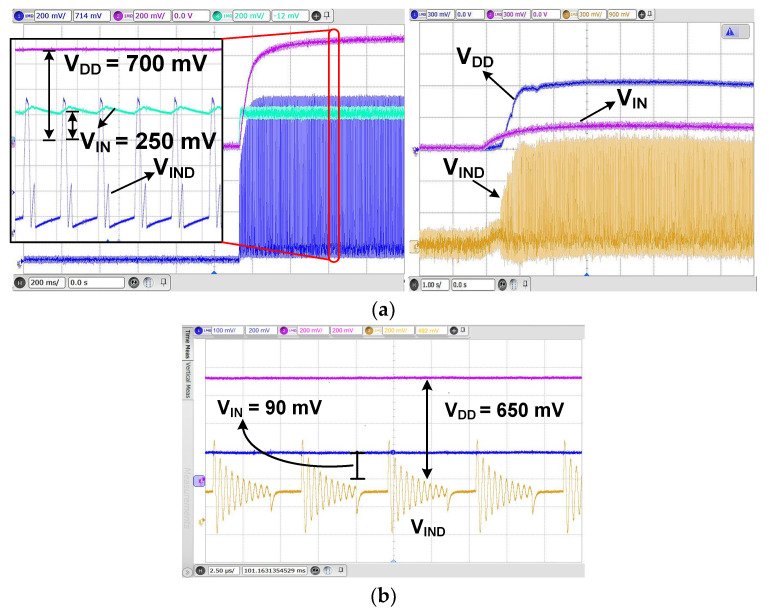
(**a**) Measured waveforms of the self-startup circuit using a power supply (left) and a commercial TEG (right). (**b**) Measured waveforms of the self-startup for *V*_IN_ = 90 mV. *R*_S_ = 30 Ω; *C*_IN_ = 0.47 μF.

Figure 15 shows the measured waveform of the oscillator. When *V*_RAMP_ is higher than *V*_REF_ = 600 mV, Φ_OSC_ is triggered high. The measured frequency of Φ_OSC_ is 44.4 kHz. Figure 16a shows the measured transient result of the self-startup. Using *V*_IN_ = 250 mV and *R*_S_ = 20 Ω, *V*_DD_ is increased to 700 mV, which is enough to enable the boost converter. The steady state is reached after 2 sec. Using the commercial TEG having *V*_IN_ = 200 mV, *V*_DD_ is increased to 600 mV, and the steady state is reached after 5 sec. These results demonstrate the successful operation of the self-startup. We further characterize the startup circuit using reduced *V*_IN_. Figure 16b shows the measured result, in which *V*_IN_ = 90 mV is increased to *V*_DD_ = 650 mV. The result shows that the gain-boosted tri-state buffer effectively reduces the minimum startup voltage. 

**Figure 17:**
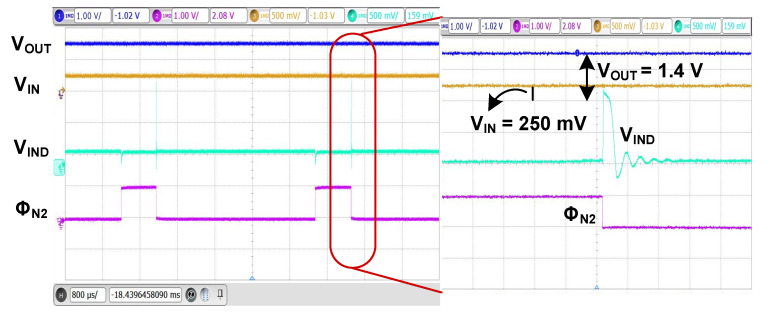
Measured waveforms of the boost converter. C_IN_ = 0.47 μF.

Figure 17 shows the measured waveforms of the converter. The experiment is performed using *V*_IN_ = 250 mV and *R*_S_ = 20 Ω. When Φ_N2_ is high, *L* is charged. When Φ_N2_ is triggered to low, the energy stored in *L* is transferred to the output. When *V*_IND_ reaches the ground, Φ_P2_ becomes high to turn off M_P2_ via the I-ZCS controller. The ringing is caused by the *LC* resonance when M_P2_ is turned off. Overall capacitance, including *C_L_*, forms resonance with *L*, and the amplitude is decayed by the path loss.

**Figure 18:**
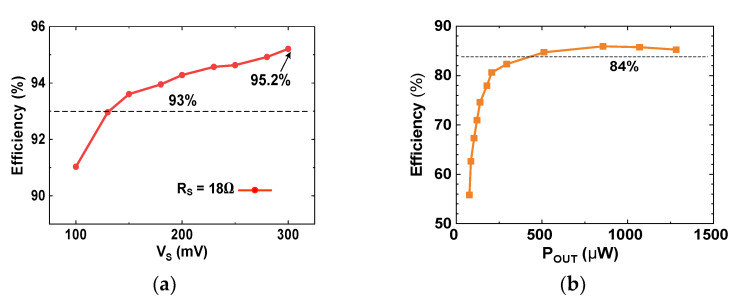
(**a**) Measured tracking efficiency of the converter; (**b**) measured conversion efficiency of the converter. *V*_IN_ = 200 mV; *R*_S_ = 18 Ω.

**Figure 19:**
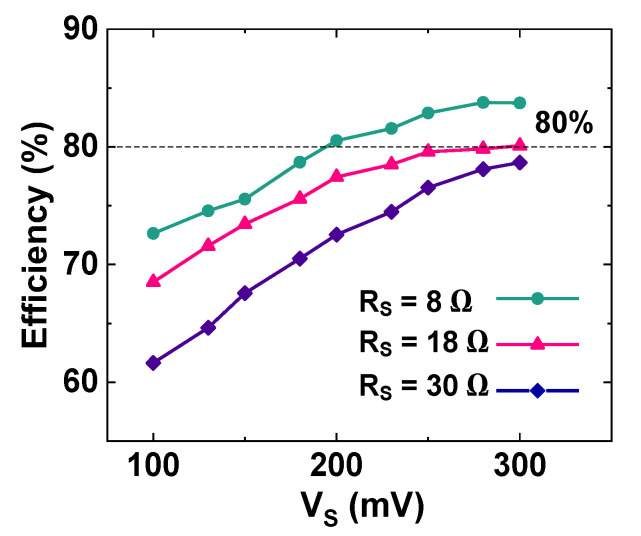
Measured end-to-end efficiency of the converter as a function of *V*_S_ for different *R*_S_. *R*_L_ = 4.7 kΩ.

Figure 18a shows the measured tracking efficiency *η*_MPPT_ = (*P*_IN_/*P*_Max_) as a function of V_S_. The results show that *η*_MPPT_ > 93% is achieved in the *V*_S_ range from 135 mV to 300 mV, with the peak *η*_MPPT_ of 95.2%. Figure 18b shows the measured conversion efficiency *η*_CONV_ = (*P*_OUT_/*P*_IN_) as a function of *P*_OUT_. *P*_OUT_ is changed from 77 μW to 1.28 mW by varying *R*_L_. The peak *η*_CONV_ = 85.9% is achieved for *P*_OUT_ = 1.07 mW. Figure 19 shows the measured *η*_EE_ = (*P*_OUT_/*P*_Max_) of the converter as a function of *V*_S_ for different *R*_S_. Because *P*_Max_ is reduced, *η*_EE_ decreases with increasing *R*_S_. The result shows that peak *η*_EE_ = 83.7% is achieved using *R*_S_ = 8 Ω and *V*_S_ = 280 mV.

Table 2 shows the comparison with previous works using the boost converter for energy harvesting [25,26,27,28,29,30,31,32]. Our approach introduces a novel technique for self-startup using the tri-state buffer, distinguishing it from the studies conducted in [26,29], which relied on a single buffer. Notably, the proposed self-startup circuit achieves a minimum startup voltage of 90 mV, outperforming all the works except [30]. Furthermore, our proposed A-MPPT and I-ZCS controllers achieve a relatively high conversion efficiency of 85.9%. In comparison, the efficiencies observed in [25,28,32] are 74.5%, 75.0%, and 25.0%, respectively. In addition, our compact design has the smallest chip area (0.03 mm^2^) among the works, including ICs implemented using the same technology node of a 28 nm CMOS [31,32]. The result shows that our work effectively handles the previous issue of bulky implementations. Consequently, our research provides practical and efficient solutions for low-cost wearable electronics and IoT applications.

## 5. Conclusions

In this paper, we propose a compact and efficient boost converter in a 28 nm CMOS for thermoelectric energy harvesting. For self-startup, we use a gain-boosted tri-state buffer in a ring oscillator to achieve a 90 mV minimum startup. A comparison with the single inverter shows that the proposed tri-state buffer achieves a 69.8% improvement in the DC gain. To extract the maximum power from the TEG, we use a FOCV-based A-MPPT controller. To remove the current sensor required by the conventional approach, we propose an I-ZCS controller to achieve an efficient DCM operation. The converter is fabricated in a 28 nm COMS process realized in a compact area of 0.03 mm^2^. The measured data show a successful I-ZCS operation using the proposed MOVT technique. Using the A-MPPT controller, the converter achieves a peak tracking efficiency of 95.2%. The converter delivers the output power of 1.07 mW with a peak conversion efficiency of 85.9%. Using the proposed techniques, TEG energy harvesting can be realized in a compact system. These characteristics are suitable for various applications, including wearable electronics, medical implants, and IoT devices.

## Figures and Tables

**Table 1:** Parameters of the power switches.

Power Switch	On-Resistance	Gate Capacitance	Size (W/L)
M_N1_	*R*_N1_ = 0.40 Ω	*C*_G,N1_ = 45.2 pF	2048 μm/35 nm
M_N2_	*R*_N2_ = 0.18 Ω	*C*_G,N2_ = 15.4 pF	2048 μm/35 nm
M_P2_	*R*_P2_ = 0.45 Ω	*C*_G,P2_ = 9.1 pF	4096 μm/35 nm

**Table 2:** Performance comparison.

	[25]	[26]	[27]	[28]	[29]	[30]	[31]	[32]	This Work
Process	65 nm	180 nm	180 nm	180 nm	180 nm	180 nm	28 nm	28 nm	28 nm
Energy source	TEG	TEG, PV	TEG	TEG, PZ	TEG	PV	TEG, PV, BFC, Battery	TEG, RF	TEG
Type	Boost	Buck-Boost	Boost	Boost	Boost	Boost	Buck-Boost	Boost	Boost
Startup technique	OSC + CP	Ring OSC + CP	CP	−	Ring OSC + CP	CP	Battery	LC OSC	Tri-state buffer, Ring OSC
Minimum startup voltage	210 mV	370 mV	500 mV	100 mV	129 mV	80 mV	−	110 mV	90 mV
ZCS technique	Dynamic comp.	gate comparators	One shot pulse gen.	Latched comp.	D- F/F	Comparator, D-F/F	Digital calibration	−	* MOVT
MPPT technique	FOCV	FOCV	^†^ AIR	^††^ DPR	OCV	^†††^ SRFG	FOCV	^††††^ ASF	FOCV
η_CONV_	74.5% @ P_OUT_ = 229 μW	−	−	75.0% @ P_OUT_ = 450 μW	−	89.0% @ P_OUT_ = 0.12 mW	89.0% @ P_OUT_ = 20 mW	25.0% @ P_OUT_ = 0.52 mW	85.9% @ P_OUT_ = 1.07 mW
η_EE_	71.5% @ V_IN_ = 240 mV	82.1% @ V_IN_ = 600 mV	82.0% @ V_IN_ = 600 mV	−	84.0% @ V_IN_ = 260 mV	86.0% @ V_IN_ = 260 mV	−	10.0% @ N/A	83.7% @ V_IN_ = 280 mV
V_OUT_	0.86–1.4 V	1.2 V	1–1.2 V	3–4 V	0.8 V	1.2 V	0.4–1.4 V	−	1.4 V
Area (mm^2^)	4.57	1.23	1.1	1.5	1.62	1.5	0.5	0.46	0.03

* Maximum output voltage tracking; ^†^ Adaptive input ripple; ^††^ Double pile-up resonance; ^†††^ Self-controlled resonant frequency generator; ^††††^ Adaptive switching frequency.

**Figure A1:**
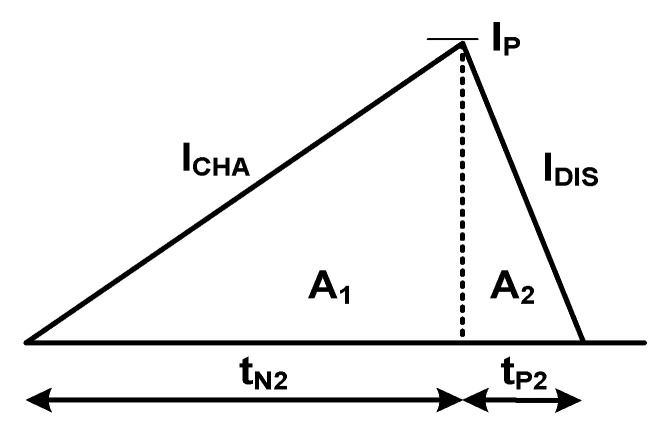
Inductor current waveform of the boost converter.

## Appendix A

Figure A1 shows the inductor current *I*_IND_ waveform of the boost converter. The *I*_IND_ is the sum of *I*_CHA_ during *t*_N2_ and *I*_DIS_ during *t*_P2_. Assuming *V*_IN_ slowly varies during the conversion, the voltage difference across *L* for *I*_CHA_ can be expressed as

(A1) $$V_{\mathrm{IN}}-0=L\frac{dI_{\mathrm{IND}}}{dt}≅L\frac{I_{P}}{t_{N2}}$$

where *I*_P_ ≈ *V*_IN_(*t*_N2_/*L*) denotes the peak current. Using the voltage across the inductor at *t*_P2_, we obtain another expression for the peak current *I*_P_:

(A2) $$I_{P}=\frac{(V_{\mathrm{OUT}}-V_{\mathrm{IN}})t_{P2}}{L}$$

The average current is obtained by integrating the current within the switching cycle and is equivalent to calculating areas *A*_1_ and *A*_2_. Using (A2), area *A*_2_ is obtained as

(A3) $$A_{2}=\frac{1}{2}t_{P2}I_{P}=\frac{1}{2L}t_{P2}^{2}(V_{\mathrm{OUT}}-V_{\mathrm{IN}})$$

Then, the output current *I*_OUT_ can be expressed as

(A4) $$I_{\mathrm{OUT}}=\frac{t_{P2}^{2}}{2L}(V_{\mathrm{OUT}}-V_{\mathrm{IN}})f_{S}$$

where *f*_S_ is the switching frequency. Using the *I*_OUT_ expression of (A4), we obtain the output resistance *R*_OUT_ as

(A5) $$R_{\mathrm{OUT}}=\frac{V_{\mathrm{OUT}}}{(V_{\mathrm{OUT}}-V_{\mathrm{IN}})f_{S}}\frac{2L}{t_{P2}^{2}}$$

Rearranging (A5) for *t*_P2_, we obtain

(A6) $$t_{P2}^{2}=\frac{2L}{R_{\mathrm{OUT}}f_{S}}\frac{V_{\mathrm{OUT}}}{\left(V_{\mathrm{OUT}}-V_{\mathrm{IN}}\right)}$$

For the present state, we use the discharging time as *t*_P2_(*n*) and the average output as *V*_OUT,AVG_(*n*). Similarly, for the previous state, we use *t*_P2_(*n*−1) and *V*_OUT,AVG_(*n*−1). Then, we obtain

(A7) $$t_{P2}^{2}(n)=\frac{2L}{R_{\mathrm{OUT}}f_{S}}\frac{V_{\mathrm{OUT},\mathrm{AVG}}(n)}{\left(V_{\mathrm{OUT},\mathrm{AVG}}(n)-V_{\mathrm{IN}}\right)}$$

(A8) $$t_{P2}^{2}(n-1)=\frac{2L}{R_{\mathrm{OUT}}f_{S}}\frac{V_{\mathrm{OUT},\mathrm{AVG}}(n-1)}{\left(V_{\mathrm{OUT},\mathrm{AVG}}(n-1)-V_{\mathrm{IN}}\right)}$$

Subtracting (A8) from (A7), we obtain

(A9) $$t_{P2}^{2}(n)-t_{P2}^{2}(n-1)=2t_{P2}\times \Delta t_{P2}=\left[\frac{V_{\mathrm{OUT},\mathrm{AVG}}(n)}{V_{\mathrm{OUT},\mathrm{AVG}}(n)-V_{\mathrm{IN}}}-\frac{V_{\mathrm{OUT},\mathrm{AVG}}(n-1)}{V_{\mathrm{OUT},\mathrm{AVG}}(n-1)-V_{\mathrm{IN}}}\right]\frac{2L}{R_{\mathrm{OUT}}f_{S}}$$

where Δ*t*_P2_ = *t*_P2_(*n*)− *t*_P2_(*n*−1). Assuming *V*_OUT_(*n*) ≈ *V*_OUT,AVG_(*n*−1) under a small-signal operation and (*V*_OUT_ ≫*V*_IN_) for the boost converter, we can simplify (A9) as

(A10) $$2t_{P2}\times \Delta t_{P2}=-\Delta V_{\mathrm{OUT}}\left(\frac{2L}{R_{\mathrm{OUT}}f_{S}}\right)\left(\frac{V_{\mathrm{IN}}}{V_{\mathrm{OUT}}^{2}}\right)$$

where Δ*V*_OUT_ = *V*_OUT,AVG_(*n*) − *V*_OUT,AVG_(*n*−1). Using (A1) and (A2), we obtain the expression for *t*_P2_ as

(A11) $$t_{P2}=t_{N2}\frac{V_{\mathrm{IN}}}{V_{\mathrm{OUT}}-V_{\mathrm{IN}}}≅t_{N2}\frac{V_{\mathrm{IN}}}{V_{\mathrm{OUT}}}$$

Using (A10) and (A11), we obtain

(A12) $$\begin{array}{l} \Delta t_{P2}=-\frac{\Delta V_{\mathrm{OUT}}}{2t_{P2}}\left(\frac{2L}{R_{\mathrm{OUT}}f_{S}}\right)\left(\frac{V_{\mathrm{IN}}}{V_{\mathrm{OUT}}^{2}}\right)=-\Delta V_{\mathrm{OUT}}\left(\frac{L}{R_{\mathrm{OUT}}f_{S}}\right)\left(\frac{V_{\mathrm{IN}}}{V_{\mathrm{OUT}}^{2}}\right)\frac{V_{\mathrm{OUT}}}{t_{N2}V_{\mathrm{IN}}}\\ \;\;\;\;\;\;\;\;=-\left(\frac{L}{t_{N2}R_{\mathrm{OUT}}f_{S}}\right)\frac{\Delta V_{\mathrm{OUT}}}{V_{\mathrm{OUT}}}. \end{array}$$

## Appendix B

The nomenclature used in this paper are listed below.

Symbol	Definition
A-MPPT	Adaptive maximum power point tracking
DCM	Discontinuous conduction mode
FOCV	Fractional open circuit voltage
I-ZCS	Indirect zero current switching
MOVT	Maximum output voltage tracking
MPPT	Maximum power point tracking
PDC	Programmable delay controller
ZCS	Zero current switching
VD	Voltage detector
LVT	Low threshold voltage
HVT	High threshold voltage
SVT	Standard threshold voltage
SLVT	Super-low threshold voltage
SC	Switched capacitor
*I*_IND_	Inductor current
*V*_TH_	Threshold voltage
*A*_V1_	Voltage gain of a single inverter
*A*_V2_	Voltage gain of the gain-boosted buffer
*P*_R,startup_	Resistive power loss of startup circuit
*P*_SW,startup_	Switching loss of startup circuit
*P*_Q,startup_	Power consumption of startup circuit
*P*_R,boost_	Resistive power loss of boost converter
*P*_SW,boost_	Switching loss of boost converter
*P*_Q,boost_	Power consumption of boost converter
*V*_OUT,AVG_(*n*)	Current average output voltage
*V*_OUT,AVG_(*n* − 1)	Previous average output voltage
